# Intrinsic and Extrinsic Neuromodulation of Olfactory Processing

**DOI:** 10.3389/fncel.2017.00424

**Published:** 2018-01-09

**Authors:** Kristyn M. Lizbinski, Andrew M. Dacks

**Affiliations:** Department of Biology, West Virginia University, Morgantown, WV, United States

**Keywords:** neuromodulation, olfaction, sensory processing, serotonin, GABA, presynaptic gain control

## Abstract

Neuromodulation is a ubiquitous feature of neural systems, allowing flexible, context specific control over network dynamics. Neuromodulation was first described in invertebrate motor systems and early work established a basic dichotomy for neuromodulation as having either an intrinsic origin (i.e., neurons that participate in network coding) or an extrinsic origin (i.e., neurons from independent networks). In this conceptual dichotomy, intrinsic sources of neuromodulation provide a “memory” by adjusting network dynamics based upon previous and ongoing activation of the network itself, while extrinsic neuromodulators provide the context of ongoing activity of other neural networks. Although this dichotomy has been thoroughly considered in motor systems, it has received far less attention in sensory systems. In this review, we discuss intrinsic and extrinsic modulation in the context of olfactory processing in invertebrate and vertebrate model systems. We begin by discussing presynaptic modulation of olfactory sensory neurons by local interneurons (LNs) as a mechanism for gain control based on ongoing network activation. We then discuss the cell-class specific effects of serotonergic centrifugal neurons on olfactory processing. Finally, we briefly discuss the integration of intrinsic and extrinsic neuromodulation (metamodulation) as an effective mechanism for exerting global control over olfactory network dynamics. The heterogeneous nature of neuromodulation is a recurring theme throughout this review as the effects of both intrinsic and extrinsic modulation are generally non-uniform.

## Introduction

Neuromodulation adjusts the biophysical and synaptic properties of neurons, allowing fine control over network dynamics (Kupfermann, 1979; Kaczmarek and Levitan, 1987; Katz, 1999). Foundational work from invertebrate motor systems (Harris-Warrick and Marder, 1991; Katz, 1995; Katz and Frost, 1995, 1996) identified two major categories of neuromodulation that modify network processing under different circumstances; intrinsic neuromodulation vs. extrinsic neuromodulation. Intrinsic neuromodulation is exerted by neurons that are within a neural network and participate in information processing undertaken by that network. The amount of intrinsic neuromodulation depends on past or ongoing network activity and provides a “memory” of the network state. Extrinsic neuromodulation is exerted by neurons that originate in independent networks and therefore provide information based on the activity of other neural networks. These can be centrifugal neurons innervating a given network or endocrine cells that release humoral factors. The amount of extrinsic neuromodulation therefore depends on the activity of other networks, rather than the network that is being modulated. Thus, extrinsic neuromodulation provides a context about the broader state of the animal. The buccal ganglion of *Aplysia* *californica*, which coordinates motor output to control biting movements, illustrates the influence of both intrinsic and extrinsic modulation within a single network (Morgan et al., 2000). The cerebral interneuron “CBI-2” initiates and directly participates in biting motor programs, making it an intrinsic element of the feeding central pattern generator (CPG). With each motor program, CBI-2 improves bite articulation via several neuromodulators (Morgan et al., 2000; Koh and Weiss, 2005, 2007; Friedman and Weiss, 2010; Dacks et al., 2012b). Biting motor programs can also be modulated based on prior exposure to food (Kupfermann, 1974) via the serotonergic metacerebral cells (MCCs) which are external to the feeding CPG. The MCCs do not initiate biting motor programs, yet their activity decreases latency of motor program initiation (Kupfermann, 1979; Kupfermann and Weiss, 1982; Morgan et al., 2000) and lesioning the MCCs reduces the frequency of biting (Rosen et al., 1989). Finally, the MCCs (the extrinsic modulators) shorten feeding motor program latency by increasing CBI-2 (the intrinsic modulator) quantal content (Proekt and Weiss, 2003). This “metamodulation” demonstrates how extrinsic modulators can target intrinsic modulatory components to exert contextual control over network activity (Katz, 1999).

While this conceptual dichotomy (intrinsic vs. extrinsic neuromodulation) has been extensively explored in motor systems, it has received less attention in sensory systems. Here, we take advantage of the depth of work on neuromodulation of olfaction to discuss mechanisms of intrinsic and extrinsic modulation within the first processing stage of the olfactory systems of mammals and insects. The intent of this review is to discuss the concepts of intrinsic and extrinsic modulation to the olfactory system and is by no means exhaustive. As an exemplar of intrinsic modulation, we discuss GABAb-mediated presynaptic inhibition as a means of gain-control modulating the dynamic range of the olfactory system based on previous and ongoing network activity. For extrinsic modulation, we discuss serotonergic inputs from outside the olfactory system that target specific neuron classes via differential receptor expression. We then discuss how serotonergic modulation of GABAergic interneurons provides an elegant mechanism by which metamodulation can use pre-existing gain control mechanisms to efficiently adjust network dynamics. Finally, we discuss the heterogeneous nature of neuromodulation throughout this review, as populations of neurons, and even the arbors of a single neuron, display a surprising degree of molecular and synaptic heterogeneity.

## Anatomy of the Olfactory System and Sources of Neuromodulation

**Figure 1:**
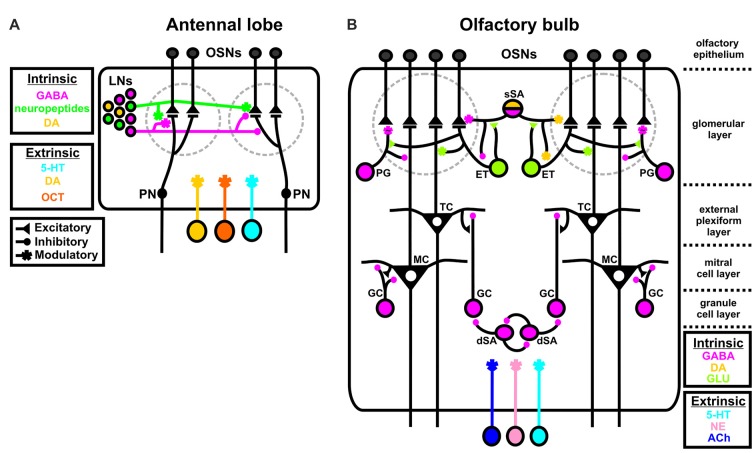
Intrinsic and extrinsic sources of neuromodulation in the insect and vertebrate olfactory system. **(A)** In the insect antennal lobe (AL), all three principal neuron types, olfactory sensory neurons (OSNs), local interneurons (LNs), and projection neurons (PNs) are subject to both intrinsic and extrinsic sources of modulation. GABA (magenta), dopamine (DA; yellow), and a suite of neuropeptides (green) released by LNs act as intrinsic modulators, while serotonin (5-HT; blue), DA, and octopamine (OCT; orange) act as extrinsic modulators to contextually alter olfactory processing. DA can be extrinsic or intrinsic depending on the species. **(B)** In the vertebrate olfactory bulb (OB), subtypes of LNs broadly serve as sources of intrinsic modulation. GABAergic periglomerular cells (PG), glutamatergic external tufted cells (ET; light green) and GABAergic/DAergic superficial short axon cells (sSA; magenta/yellow) synapse onto OSNs, mitral and tufted cells (M/Ts) and each other in the glomerular layer. GABAergic granule cells (GC) synapse onto M/Ts to alter OB output and GABAergic deep short axon cells (dSA) both reciprocally synapse onto themselves and GCs. Both the AL and OB are innervated by extrinsic sources of 5-HT, norephinephrine (NE; pink), and acetylcholine (ACh; dark purple). The “~” symbol at the PG to OSN synapse indicates that this is a non-traditional synapse that depends upon GABA spillover.

There are many parallels between the insect and vertebrate olfactory systems (Hildebrand and Shepherd, 1997; Ache and Young, 2005). Notably, for this review, both are subject to a broad suite of neuromodulators. In the insect antennal lobe (AL), odorants bind to chemosensory proteins expressed by input neurons called olfactory sensory neurons (OSNs; Figure 1A). Individual OSNs generally express a single chemosensory receptor protein (Vosshall et al., 1999; Vosshall, 2000; Goldman et al., 2005; Joseph and Carlson, 2015) and all OSNs that express the same chemosensory receptor protein project into the same sub-structure in the AL called a glomerulus. Within a glomerulus, OSNs synapse upon output neurons called projection neurons (PNs). PNs then send olfactory information to higher order brain centers like the mushroom bodies (involved in learning/memory; reviewed in Zars, 2000; Owald and Waddell, 2015) and the lateral horn (involved in odor valence; Gupta and Stopfer, 2011; Sachse and Beshel, 2016; Schultzhaus et al., 2017). Finally, a diverse population of local interneurons (LNs; Seki and Kanzaki, 2008; Chou et al., 2010; Seki et al., 2010; Reisenman et al., 2011) refines the input/output relationship of OSNs and PNs. All three principal neuron types, OSNs, LNs and PNs, are subject to both intrinsic and extrinsic sources of neuromodulation. LNs release GABA, dopamine (DA) and a suite of neuropeptides (Homberg et al., 1990; Kirchhof et al., 1999; Berg et al., 2007; Utz et al., 2008; Carlsson et al., 2010; Chou et al., 2010; Siju et al., 2014; Fusca et al., 2015; Hamanaka et al., 2016; Tedjakumala et al., 2017), while the AL is innervated by centrifugal neurons releasing serotonin (5-HT), DA and octopamine (OCT) which act as extrinsic modulators (Kent et al., 1987; Rehder et al., 1987; Salecker and Distler, 1990; Ignell, 2001; Dacks et al., 2005, 2006, 2012a; Sinakevitch et al., 2005; Sinakevitch and Strausfeld, 2006).

Similarly, in the vertebrate olfactory system odorants activate OSNs in the olfactory epithelium which project into glomeruli in the olfactory bulb (OB; Figure 1B). OSNs synapse onto two types of output neurons called mitral and tufted (M/T) cells, which send olfactory information in part to the piriform cortex, olfactory tubercle and other secondary targets. Much like insects, the OB relies on heterogeneous populations of LNs to refine M/T cell output. Subtypes of LNs in the glomerular layer (“juxtaglomerular neurons”) exhibit complex connectivity with the major OB cell types (Wachowiak and Shipley, 2006). Juxtaglomerular neurons can be subdivided into three classes. GABAergic periglomerular cells (PG) synapse onto M/T cells and have an unconventional inhibitory relationship with OSNs in which PG cells may influence OSNs via GABAergic spillover and not via a traditional inhibitory synapse (Pinching and Powell, 1971; Aroniadou-Anderjaska et al., 2000; Wachowiak et al., 2005). Glutamatergic external tufted cells (ET) synapse onto PG cells, M/T cells and superficial short axon cells (sSA). There is evidence that subsets of PG and ET cells may also release DA (Kosaka and Kosaka, 2016). Finally, GABAergic/DAergic sSA cells synapse onto OSNs, and ETs, and widely interconnect both neighboring and distant glomeruli in the glomerular layer (Aungst et al., 2003; Kiyokage et al., 2010; Liu et al., 2013). In the granule cell (GC) layer of the OB, GABAergic GCs provide feedback inhibition onto M/T cells (Shepherd et al., 2007; Burton, 2017). Furthermore, GABAergic deep short axon cells (dSA; Eyre et al., 2008; Burton et al., 2017) synapse onto themselves and reciprocally synapse upon GCs (Burton, 2017). PG and ET cells also appear to express a wide variety of neuropeptides, including NPY, VIP and CCK (Seroogy et al., 1985; Gall et al., 1986). The OB is also subject to extrinsic sources of modulation including 5-HT, norepinephrine (NE) and acetylcholine (ACh), that are released from centrifugal neurons outside of the OB (McLean and Shipley, 1987; Linster and Cleland, 2002, 2016; Kiselycznyk et al., 2006; Matsutani and Yamamoto, 2008; Fletcher and Chen, 2010; Steinfeld et al., 2015).

## Intrinsic Modulation as a Means of Presynaptic Gain Control

The olfactory system must efficiently encode odorant information over a wide concentration range to produce reliable representations of odor identity. Heterogeneous populations of LNs and juxtaglomerular neurons alter the input/output relationship between principal cell types to accomplish much of this computation. LNs can release a variety of transmitters including many neuromodulators that act over a range of timescales and in *Drosophila*, lateral input from inhibitory LNs scales with overall network activation (Olsen and Wilson, 2008). Thus, LNs intrinsically modulate odor coding within the context of current and previous network activation. Intrinsic modulation of olfactory processing can: (1) alter network output based on the strength of odor input; (2) mediate long-lasting temporal effects via metabotropic receptors; and (3) regulate the dynamic range of output neurons, allowing for reliable coding of odor identity across a range of stimulus intensities.

**Figure 2:**
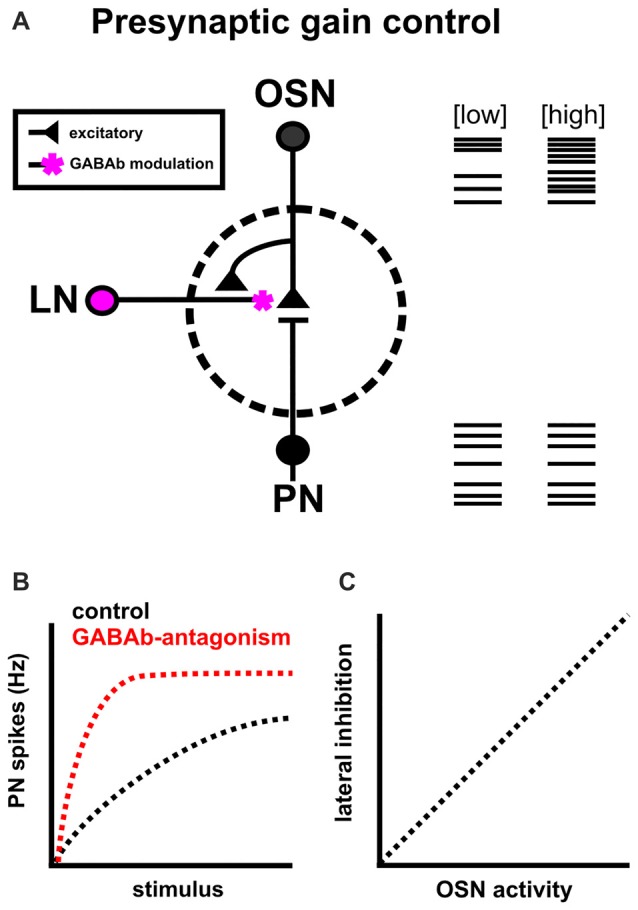
Intrinsic modulation as a means of presynaptic gain control. **(A)** Presynaptic gain control alters the signal strength between OSNs and PNs to filter out noise from spontaneous firing of OSNs, avoid PN saturation and expand the dynamic range of PNs. It does this by reducing presynaptic calcium levels in OSNs via GABAb receptors, and reducing the likelihood of acetycholine release onto PNs. **(B)** GABAb blockade (red) increases presynaptic calcium influx and decreases the range of OSN input over which PN firing rate can change, ultimately resulting in PN saturation (based on results from Root et al., 2008; Olsen et al., 2010). **(C)** GABAergic lateral inhibition scales with network activation (as measured by OSN activity; Olsen and Wilson, 2008).

The information transferred from individual OSNs to PNs is highly reliable (Murphy et al., 2004) yet non-linear (Wilson et al., 2004; Bhandawat et al., 2007; Olsen and Wilson, 2008). At high stimulus intensities, output neuron activity can saturate such that further increases in OSN output do not result in a concomitant increase in output neuron activity. To avoid saturation, the olfactory system relies on presynaptic inhibition as a means of gain control. Gain control adjusts the signal strength between input and output neurons to filter out noise from spontaneous firing of OSNs (Wilson, 2013), avoid saturation (Martin et al., 2011) and adjust the dynamic range of output neurons (Olsen and Wilson, 2008; Root et al., 2008). It does this, broadly, by activating GABAb receptors on presynaptic terminals to reduce presynaptic calcium levels, resulting in reduced transmitter release from the presynaptic neuron (Figure 2A; Wang, 2012). While early studies on GABAergic inhibition in the insect AL focused on the role of ionotropic GABAa mediated lateral inhibition (Waldrop et al., 1987; Christensen et al., 1993, 1998a,b; Lei et al., 2002), GABAa blockade in insects does not fully block a slower form of inhibition (MacLeod and Laurent, 1996; Christensen et al., 1998b; Bazhenov et al., 2001; Wilson et al., 2004; Wilson and Laurent, 2005). A slower GABA component had also been suggested as GABAb receptor agonists reduce the responses of M/T cells responses to olfactory nerve stimulation (Nickell et al., 1994). Together this suggested that: (1) GABA may also act presynaptically; and (2) a slower, metabotropic mechanism is at play.

In *Drosophila melanogaster*, GABAb blockade on OSN terminals increases presynaptic calcium influx, broadens odor tuning of PNs and decreases the range of OSN input over which PN firing rate can change, ultimately resulting in PN saturation (Figure 2B; Olsen and Wilson, 2008; Root et al., 2008). In normal conditions, PN responses are normalized via increased lateral inhibition which scales with ORN activity (Figure 2C; Olsen et al., 2010). Overall, this suggests that interglomerular presynaptic inhibition adjusts the dynamic range of PNs to avoid saturation and refines the breadth of odor tuning across a wide range of stimulus intensities (Wang, 2012). It is important to note that GABA is not the sole modulator of gain control in this system, as the neuropeptides tachykinin (Ignell et al., 2009), and short neuropeptide F (Root et al., 2011; Ko et al., 2015) also mediate presynaptic inhibition.

In the OB, electron microscopy and anatomical studies in rats revealed metabotropic GABAb and D_2_ receptor expression on vertebrate OSNs (Bonino et al., 1999; Koster et al., 1999) and direct physiological evidence demonstrated that DA modulates the olfactory nerve synapse (Hsia et al., 1999; Berkowicz and Trombley, 2000) and that presynaptic inhibition is mediated by GABAb receptors (Aroniadou-Anderjaska et al., 2000). Broadly, presynaptic inhibition suppresses calcium influx at OSN axon terminals (Wachowiak and Cohen, 1998, 1999) via both GABAb (Aroniadou-Anderjaska et al., 2000; Wachowiak et al., 2005) and D_2_ receptor activation (Ennis et al., 2001; Vaaga et al., 2017), potentially decreasing M/T firing rates. Additionally, GABAergic presynaptic inhibition appears to have both a tonic component that is consistent across stimulus strength, as well as a feedback component that alters glomerular input in an activity dependent manner (Pirez and Wachowiak, 2008). There are a few hypothesized roles for presynaptic inhibition in the OB: (1) that it functions as an adaptive gain control mechanism (Nickell et al., 1994; McGann et al., 2005; Vučinić et al., 2006; Wachowiak and Shipley, 2006; Banerjee et al., 2015; Vaaga et al., 2017); (2) it suppresses OSN input during sniffing (Aroniadou-Anderjaska et al., 2000; reviewed in Wachowiak and Shipley, 2006); and (3) it sharpens the representations of odors across the glomerular map (Vučinić et al., 2006).

However, it is still unclear whether presynaptic inhibition mediates gain control in the OB. A hallmark of gain control is that inhibition is stronger at higher stimulus intensities and weaker at lower stimulus intensities (Robinson and McAlpine, 2009; Saalmann and Kastner, 2009; Martin et al., 2011; Wang, 2012). To demonstrate that presynaptic inhibition adjusts the dynamic range of OB output, presynaptic inhibition must scale with odor concentration. One study found that both weak and strong odor-evoked inputs are subject to the same amount of GABAergic inhibition, and tonic inhibition does not scale with the strength of OSN activation (Pirez and Wachowiak, 2008). This suggests that presynaptic inhibition may alter OSN sensitivity, rather than adjust the dynamic range of M/T cells. Another study demonstrated that juxtaglomerular GABA/DAergic cells, likely sSA cells, exert concentration dependent gain control onto M/T cells (Banerjee et al., 2015). However, it is unclear whether this gain control has a presynaptic component, as this study focused on the synaptic interactions of ET, sSA and M/T cells. Finally, activation of GABA/DAergic sSA cells inhibit presynaptic OSNs, resulting in decreased spiking in M/T cells, and M/T attenuation is blocked by GABAb and D_2_ receptor antagonists (Vaaga et al., 2017). However, this does not rule out a GABAb-dependent postsynaptic mechanism of gain control, as sSA activation could act via multiple synapses to control M/T output. Finally, it is still unclear which exact subpopulation(s) of neurons mediate presynaptic inhibition. Potential sources include GABA spillover from GABAergic PG neurons (Aroniadou-Anderjaska et al., 2000; Wachowiak et al., 2005), direct inhibition of OSNs via GABA from sSA (Vaaga et al., 2017), or altered glutamatergic ET cell activity to indirectly influence OB output (Pirez and Wachowiak, 2008; Banerjee et al., 2015).

### Local Interneuron Heterogeneity

While presynaptic inhibition is ubiquitous, it is not exerted evenly across the olfactory system. In *Drosophila*, some glomeruli are more subject to inhibition than others simply due to differences in glomerulus-specific LN innervation (Wilson and Laurent, 2005; Chou et al., 2010) and OSN GABAb receptor expression (Root et al., 2008). This suggests that specific odors differ in the amount of “shelter” they need from ongoing activity in the olfactory system, and are therefore insulated from presynaptic gain control. Furthermore, LNs and juxtaglomerular neurons are heterogeneous in their morphology, physiology and transmitter content (Seki and Kanzaki, 2008; Carlsson et al., 2010; Chou et al., 2010; Seki et al., 2010; Reisenman et al., 2011; Nagayama et al., 2014). Consequently, this heterogeneity has made it challenging to determine the sub-populations of neurons involved and the mechanisms by which they mediate presynaptic gain control. For example, individual LNs in the moth *Manduca sexta* likely express up to five transmitters, and co-expression of neuropeptides is variable across the entire population (Lizbinski et al., 2017). Thus, few LNs expresses the same combination of transmitters, resulting a dynamic cocktail of neuromodulators that regulate the modulatory tone of the network. Overall this suggests that while LNs and juxtaglomerular neurons function as intrinsic modulators of olfactory coding, a variety of mechanisms make their influence non-uniform.

## Extrinsic Modulation of Olfactory Processing

Animals must constantly adjust their sensory processing to meet the ongoing demands of a dynamic internal and external environment. Both insects and vertebrates heavily rely on their sense of smell to find mates, acquire food and avoid harmful threats in their environment. However, the relative importance of different odors varies with current physiological demands. Extrinsic modulatory neurons from other networks can therefore adjust activity within the OB and AL to provide the context of current internal demands of the individual animal. The olfactory system is subject to a number of extrinsic sources of neuromodulation including 5-HT, DA (in some insects), ACh and NE that have been associated with broad physiological states like waking state, aversion, attention and learning/memory (McLean and Shipley, 1987; Mandairon et al., 2006; Matsutani and Yamamoto, 2008; Fletcher and Chen, 2010; Wasserman et al., 2013; but see Linster and Cleland, 2016). Here, we will focus on the effects of 5-HT as both the OB and AL receive 5-HT innervation from extrinsic sources, and there are many similarities between the cellular and molecular features of serotonergic modulation in both networks.

### Cell Class Specific Effects of Serotonergic Modulation

In the AL and OB, neuronal class specific 5-HT receptor expression results in relatively heterogeneous effects of 5-HT, even within the same neuronal class. The OB, and in particular, the glomerular layer, receives serotonergic innervation from a large number of Median and Dorsal Raphe neurons (Pinching and Powell, 1971; McLean and Shipley, 1987; Shipley and Ennis, 1996; Gómez et al., 2005; Steinfeld et al., 2015; Suzuki et al., 2015; Muzerelle et al., 2016) and each AL across a wide range of insects typically receives input from one to two serotonergic neurons (Kent et al., 1987; Salecker and Distler, 1990; Wegerhoff, 1999; Ignell, 2001; Dacks et al., 2006; Roy et al., 2007). However, despite the ubiquity of 5-HT in the olfactory systems across taxa, the consequences of serotonergic modulation of olfaction have been remarkably uneven across model systems and behavioral tasks. Pharmacological studies have suggested that 5-HT facilitates odor preference learning in rat pups (McLean et al., 1993, 1996; Langdon et al., 1997; Price et al., 1998; Yuan et al., 2003) and enhances behavioral attraction to sex pheromone in moths (Linn and Roelofs, 1986; Gatellier et al., 2004; Kloppenburg and Mercer, 2008). This work suggests that 5-HT upregulates olfactory sensitivity. However, studies directly manipulating serotonergic neurons or serotonergic signaling in the olfactory system indicate that the role of 5-HT is more complex. For instance, conditionally eliminating tryptophan hydroxylase 2 expression in the raphe of mice, and therefore 5-HT synthesis after olfactory development, had no effect on performance in several general olfactory behavioral assays (Carlson et al., 2016). In *Drosophila*, suppressing the activity of the serotonergic neurons in the AL (the “CSDns”) increases CO_2_ avoidance, while blocking synaptic transmission decreases sensitivity to the pheromone cVA (Singh et al., 2013), suggesting that the effects of 5-HT can be odor dependent. Furthermore, the CSDns modulate ethanol attraction in concert with other serotonergic neurons that do not innervate the AL (Xu et al., 2016).

Similar to behavioral studies, the physiological effects of 5-HT within the olfactory system are also heterogeneous. Early studies in the rabbit OB showed that application of 5-HT decreased spontaneous firing rate of mitral cells (MCs; Bloom et al., 1964). More recent studies in rats revealed that 5-HT can directly (via the 5-HT2a receptor) and indirectly (via 5-HT2a receptor expression in ETs) excite MCs, yet also increases inhibition exerted upon MCs by depolarizing a subset of juxtaglomerular cells (via 5-HT2c; Hardy et al., 2005; Petzold et al., 2009; Liu et al., 2012; Schmidt and Strowbridge, 2014; Brunert et al., 2016; Huang et al., 2017). Stimulating Raphe input specifically to the OB depolarizes tufted cells (TCs; Kapoor et al., 2016) and has a heterogeneous effect on MC baseline activity (Brunert et al., 2016; Kapoor et al., 2016). However, as discussed below, dual-transmission of glutamate and serotonin by Raphe neurons complicates these findings (Liu et al., 2014). Raphe stimulation also enhances PG and sSA cell (Brunert et al., 2016) and TC responses (Kapoor et al., 2016) to clean and odor laden air. Raphe stimulation appears to predominantly enhance MC odor-evoked responses (Brunert et al., 2016), however this can be odor dependent (Kapoor et al., 2016). Consistent with a theme of heterogeneity, 5-HT was recently demonstrated to excite MCs in the main OB (MOB), yet inhibit MCs in the accessory OB (AOB; Huang et al., 2017). In moths, bath applied 5-HT reduces two K^+^ conductances (Mercer et al., 1995, 1996; Kloppenburg et al., 1999), enhancing PN and LN excitability resulting in increased odor evoked activity (Kloppenburg et al., 1999; Dacks et al., 2008). However, this only occurs for roughly half of the neurons recorded, and in some instances 5-HT decreases odor evoked responses in an odor-dependent manner (Kloppenburg et al., 1999; Dacks et al., 2008). In *Drosophila*, bath application of 5-HT enhances PN odor-evoked responses and sensitivity (Dacks et al., 2009; Zhang and Gaudry, 2016). However, pharmacological manipulations demonstrate that endogenous 5-HT reduces PN odor-evoked responses in the AL (Zhang and Gaudry, 2016). Surprisingly, the sole source of serotonergic innervation to the AL (the CSDns) do not affect PN responses to cVA, yet other serotonergic neurons outside the AL do affect cVA responses (Zhang and Gaudry, 2016). These results suggest that the AL can be modulated by both synaptic and non-synaptic sources of 5-HT, perhaps via the hemeolymph, in an odor-dependent manner.

The heterogeneous effects of 5-HT in the olfactory system likely arise due to cell-type specific 5-HT receptor expression and the heterogeneity of serotonergic neurons innervating the AL and OB. There are at least ten 5-HT receptors expressed in the OB (Appel et al., 1990; Hellendall et al., 1993; Shen et al., 1993; Tecott et al., 1993; Watts et al., 1994; McLean et al., 1995; Wright et al., 1995; Waeber et al., 1998; Bai et al., 2004; Lucaites et al., 2005; Petzold et al., 2009) and all five insect 5-HT receptors are expressed in the ALs of *Drosophila* (Sizemore and Dacks, 2016) and *Manduca* (Dacks et al., 2013). In *Drosophila*, each neuronal class expresses a different combination of 5-HT receptors. However, any given receptor is only expressed by a subset of neurons within that class (Sizemore and Dacks, 2016) which likely contributes to the non-uniform effects of 5-HT. Similarly, 5-HT directly enhances MOB MCs via the 5-HT2A receptor, yet inhibits AOB MCs via both the 5-HT1 receptor and enhanced GABAergic transmission to MCs due to 5-HT2 receptor expression by interneurons (Huang et al., 2017). Consequently, complex receptor expression patterns likely play a major role in the observed heterogeneity in the effects of 5-HT. Heterogeneity of serotonergic neurons also likely contribute to the non-uniform effects of 5-HT in the olfactory system. Raphe neurons can release both 5-HT and glutamate (Liu et al., 2014) and only glutamate receptor antagonists block the Raphe-induced depolarization of TCs (Kapoor et al., 2016). Serotonergic neurons innervate different functional zones within the OB (Won et al., 1998; Gómez et al., 2005; Steinfeld et al., 2015) and even different functional zones within glomeruli in *Manduca* (Sun et al., 1993; Lizbinski et al., 2016). Glomerular specific differences in serotonergic innervation have also been observed in the OB (Gómez et al., 2005), and the processes of the CSDns in *Drosophila* (Singh et al., 2013). Furthermore, the distribution of CSDn active zones vary widely across glomeruli, yet are highly stereotyped across individual animals (Coates et al., 2017). Thus, even within a single identified modulatory neuron, specific traits can be heterogeneous across compartments. In addition, the CSDns receive network wide inhibition from LNs and glomerulus-specific excitation from OSNs and PNs, indicating that 5-HT modulation cannot be considered purely “Top-Down” (Coates et al., 2017). Since the CSDns receive input based on AL network dynamics as well as from other networks, they can be considered partially intrinsic to the AL.

Finally, the circumstances in which 5-HT is released are surprisingly varied (Andrade and Haj-Dahmane, 2013; Dayan and Huys, 2015). The levels of 5-HT in the AL of moths fluctuate throughout the day, peaking when moths are most active (Kloppenburg et al., 1999) reminiscent of daily fluctuations of Raphe neuron activity and 5-HT production (Trulson and Jacobs, 1979; Jacobs and Fornal, 1991; Park et al., 1999; Corthell et al., 2013). Raphe neurons also have a relatively heterogeneous transcriptional profile (Okaty et al., 2015) and individual Raphe neurons can respond to either reward or punishing stimuli (Nakamura et al., 2008; Ranade and Mainen, 2009; Bromberg-Martin et al., 2010; Miyazaki et al., 2011a,b; Nakamura, 2013; Liu et al., 2014; Pollak Dorocic et al., 2014; Weissbourd et al., 2014; Cohen et al., 2015; Hayashi et al., 2015; Li et al., 2016; Luo et al., 2016), as well as display experience dependent plasticity in response properties (Zhong et al., 2017). The heterogeneous nature of serotonergic neurons and the complicated context in which 5-HT is released likely contribute to the non-uniform effects that have been observed for the physiological and behavioral consequences of 5-HT.

## Metamodulation: Extrinsic Modulation of Intrinsic Modulation

Metamodulation, or the modulation of modulation, allows extrinsic neurons to exert global control over already existing intrinsic modulatory circuits (Katz, 1999). Centrifugal neurons that innervate the olfactory system often target LNs and juxtaglomerular neurons (Matsutani and Yamamoto, 2008; Mouret et al., 2009) as an efficient mechanism for altering network processing. Presynaptic inhibition provides powerful, odor-specific control over the dynamic output of the olfactory system. Some studies have suggested that this mechanism may be further modulated via extrinsic inputs in the context of behavioral state (Pirez and Wachowiak, 2008; McGann, 2013). Specifically, widely projecting serotonergic neurons (as detailed above) target sub-populations of LNs to indirectly influence presynaptic activity of OSNs and alter stimulus dependent inhibition. In insects, 5-HT increases lateral GABAergic input to OSNs to adjust GABAb mediated presynaptic gain control (Dacks et al., 2009). In the OB, 5-HT depolarizes ETs via the 5-HT2a receptor, sSAs via 5-HT2c, and indirectly excites both sSAs and PGs via glutamatergic ETs. This increases GABAergic/DAergic modulation onto presynaptic terminals of OSNs, reduces OSN output, and ultimately may reduce M/T firing rate to alter OB output (Petzold et al., 2009; Liu et al., 2012; Brill et al., 2016). Overall, these studies suggest that extrinsic serotonergic modulation exerts global control over olfactory network dynamics, in part, by targeting an intrinsic modulatory network.

## Conclusion

1. Intrinsic presynaptic inhibition expands the dynamic range of output neurons, allowing the olfactory system to encode odors across a wide range of concentrations.
2. Extrinsic modulation adjusts olfactory processing in the AL and OB based on the activity of other neural networks.
3. The integration of both intrinsic and extrinsic neuromodulation merges both history of network activation with global context of physiological state to adjust the broad modulatory tone of the olfactory system as a whole.
4. Intrinsic and extrinsic modulatory mechanisms exert a heterogeneous influence due to complex patterns of modulatory receptor expression, cell-to-cell variability and complex connectivity in the olfactory system.

## Author Contributions

KML and AMD both conceived of the ideas for this review and co-wrote the manuscript. Author order was determined by degree of caffeination.

## Conflict of Interest Statement

The authors declare that the research was conducted in the absence of any commercial or financial relationships that could be construed as a potential conflict of interest.

## References

[B1] Ache B. W. Young J. M. (2005). Olfaction: diverse species, conserved principles. Neuron 48, 417–430. 10.1016/j.neuron.2005.10.022 16269360

[B2] Andrade R. Haj-Dahmane S. (2013). Serotonin neuron diversity in the dorsal raphe. ACS Chem. Neurosci. 4, 22–25. 10.1021/cn300224n 23336040 PMC3547477

[B3] Appel N. M. Mitchell W. M. Garlick R. K. Glennon R. A. Teitler M. De Souza E. B. (1990). Autoradiographic characterization of (+-)-1–(2,5-dimethoxy-4–[125I] iodophenyl)-2-aminopropane ([125I]DOI) binding to 5-HT2 and 5-HT1c receptors in rat brain. J. Pharmacol. Exp. Ther. 255, 843–857. 2243353

[B4] Aroniadou-Anderjaska V. Zhou F. M. Priest C. A. Ennis M. Shipley M. T. (2000). Tonic and synaptically evoked presynaptic inhibition of sensory input to the rat olfactory bulb via GABA_B_ heteroreceptors. J. Neurophysiol. 84, 1194–1203. 10.1152/jn.2000.84.3.1194 10979995

[B5] Aungst J. L. Heyward P. M. Puche A. C. Karnup S. V. Hayar A. Szabo G.. (2003). Centre-surround inhibition among olfactory bulb glomeruli. Nature 426, 623–629. 10.1038/nature02185 14668854

[B6] Bai F. Yin T. Johnstone E. M. Su C. Varga G. Little S. P.. (2004). Molecular cloning and pharmacological characterization of the guinea pig 5-HT1E receptor. Eur. J. Pharmacol. 484, 127–139. 10.1016/j.ejphar.2003.11.019 14744596

[B7] Banerjee A. Marbach F. Anselmi F. Koh M. S. Davis M. B. Garcia da Silva P.. (2015). An interglomerular circuit gates glomerular output and implements gain control in the mouse olfactory bulb. Neuron 87, 193–207. 10.1016/j.neuron.2015.06.019 26139373 PMC4633092

[B8] Bazhenov M. Stopfer M. Rabinovich M. Abarbanel H. D. Sejnowski T. J. Laurent G. (2001). Model of cellular and network mechanisms for odor-evoked temporal patterning in the locust antennal lobe. Neuron 30, 569–581. 10.1016/s0896-6273(01)00286-0 11395015 PMC2907737

[B9] Berg B. G. Schachtner J. Utz S. Homberg U. (2007). Distribution of neuropeptides in the primary olfactory center of the heliothine moth Heliothis virescens. Cell Tissue Res. 327, 385–398. 10.1007/s00441-006-0318-x 17013588

[B10] Berkowicz D. A. Trombley P. Q. (2000). Dopaminergic modulation at the olfactory nerve synapse. Brain Res. 855, 90–99. 10.1016/s0006-8993(99)02342-2 10650134

[B11] Bhandawat V. Olsen S. R. Gouwens N. W. Schlief M. L. Wilson R. I. (2007). Sensory processing in the *Drosophila* antennal lobe increases reliability and separability of ensemble odor representations. Nat. Neurosci. 10, 1474–1482. 10.1038/nn1976 17922008 PMC2838615

[B12] Bloom F. E. Costa E. Salmoiraghi G. C. (1964). Analysis of individual rabbit olfactory bulb neuron responses to the microelectrophoresis of acetylcholine, norepinephrine and serotonin synergists and antagonists. J. Pharmacol. Exp. Ther. 146, 16–23. 14221220

[B13] Bonino M. Cantino D. Sassoè-Pognetto M. (1999). Cellular and subcellular localization of γ-aminobutyric acid_B_ receptors in the rat olfactory bulb. Neurosci. Lett. 274, 195–198. 10.1016/s0304-3940(99)00697-7 10548423

[B14] Brill J. Shao Z. Puche A. C. Wachowiak M. Shipley M. T. (2016). Serotonin increases synaptic activity in olfactory bulb glomeruli. J. Neurophysiol. 115, 1208–1219. 10.1152/jn.00847.2015 26655822 PMC4808087

[B15] Bromberg-Martin E. S. Hikosaka O. Nakamura K. (2010). Coding of task reward value in the dorsal raphe nucleus. J. Neurosci. 30, 6262–6272. 10.1523/JNEUROSCI.0015-10.2010 20445052 PMC3467971

[B16] Brunert D. Tsuno Y. Rothermel M. Shipley M. T. Wachowiak M. (2016). Cell-type-specific modulation of sensory responses in olfactory bulb circuits by serotonergic projections from the raphe nuclei. J. Neurosci. 36, 6820–6835. 10.1523/JNEUROSCI.3667-15.2016 27335411 PMC4916254

[B17] Burton S. D. (2017). Inhibitory circuits of the mammalian main olfactory bulb. J. Neurophysiol. 118, 2034–2051. 10.1152/jn.00109.2017 28724776 PMC5626887

[B18] Burton S. D. LaRocca G. Liu A. Cheetham C. E. Urban N. N. (2017). Olfactory bulb deep short-axon cells mediate widespread inhibition of tufted cell apical dendrites. J. Neurosci. 37, 1117–1138. 10.1523/JNEUROSCI.2880-16.2016 28003347 PMC5296792

[B20] Carlsson M. A. Diesner M. Schachtner J. Nässel D. R. (2010). Multiple neuropeptides in the *Drosophila* antennal lobe suggest complex modulatory circuits. J. Comp. Neurol. 518, 3359–3380. 10.1002/cne.22405 20575072

[B19] Carlson K. S. Whitney M. S. Gadziola M. A. Deneris E. S. Wesson D. W. (2016). Preservation of essential odor-guided behaviors and odor-based reversal learning after targeting adult brain serotonin synthesis. eNeuro 3:ENEURO.0257-16.2016. 10.1523/ENEURO.0257-16.2016 27896310 PMC5112565

[B21] Chou Y. H. Spletter M. L. Yaksi E. Leong J. C. Wilson R. I. Luo L. (2010). Diversity and wiring variability of olfactory local interneurons in the *Drosophila* antennal lobe. Nat. Neurosci. 13, 439–449. 10.1038/nn.2489 20139975 PMC2847188

[B22] Christensen T. A. Waldrop B. R. Harrow I. D. Hildebrand J. G. (1993). Local interneurons and information processing in the olfactory glomeruli of the moth Manduca sexta. J. Comp. Physiol. A 173, 385–399. 10.1007/bf00193512 8254565

[B23] Christensen T. A. Waldrop B. R. Hildebrand J. G. (1998a). GABAergic mechanisms that shape the temporal response to odors in moth olfactory projection neurons. Ann. N Y Acad. Sci. 855, 475–481. 10.1111/j.1749-6632.1998.tb10608.x 9929641

[B24] Christensen T. A. Waldrop B. R. Hildebrand J. G. (1998b). Multitasking in the olfactory system: context-dependent responses to odors reveal dual GABA-regulated coding mechanisms in single olfactory projection neurons. J. Neurosci. 18, 5999–6008. 9671685 10.1523/JNEUROSCI.18-15-05999.1998 PMC6793051

[B25] Coates K. E. Majot A. T. Zhang X. Michael C. T. Spitzer S. L. Gaudry Q.. (2017). Identified serotonergic modulatory neurons have heterogeneous synaptic connectivity within the olfactory system of *Drosophila*. J. Neurosci. 37, 7318–7331. 10.1523/JNEUROSCI.0192-17.2017 28659283 PMC5546105

[B26] Cohen J. Y. Amoroso M. W. Uchida N. (2015). Serotonergic neurons signal reward and punishment on multiple timescales. Elife 4:e06346. 10.7554/eLife.06346 25714923 PMC4389268

[B27] Corthell J. T. Stathopoulos A. M. Watson C. C. Bertram R. Trombley P. Q. (2013). Olfactory bulb monoamine concentrations vary with time of day. Neuroscience 247, 234–241. 10.1016/j.neuroscience.2013.05.040 23727009 PMC3722297

[B28] Dacks A. M. Christensen T. A. Agricola H. J. Wollweber L. Hildebrand J. G. (2005). Octopamine-immunoreactive neurons in the brain and subesophageal ganglion of the hawkmoth Manduca sexta. J. Comp. Neurol. 488, 255–268. 10.1002/cne.20556 15952164 PMC1363738

[B29] Dacks A. M. Christensen T. A. Hildebrand J. G. (2006). Phylogeny of a serotonin-immunoreactive neuron in the primary olfactory center of the insect brain. J. Comp. Neurol. 498, 727–746. 10.1002/cne.21076 16927264

[B30] Dacks A. M. Christensen T. A. Hildebrand J. G. (2008). Modulation of olfactory information processing in the antennal lobe of Manduca sexta by serotonin. J. Neurophysiol. 99, 2077–2085. 10.1152/jn.01372.2007 18322001

[B31] Dacks A. M. Green D. S. Root C. M. Nighorn A. J. Wang J. W. (2009). Serotonin modulates olfactory processing in the antennal lobe of *Drosophila*. J. Neurogenet. 23, 366–377. 10.3109/01677060903085722 19863268 PMC2850205

[B32] Dacks A. M. Reale V. Pi Y. Zhang W. Dacks J. B. Nighorn A. J.. (2013). A characterization of the manduca sexta serotonin receptors in the context of olfactory neuromodulation. PLoS One 8:e69422. 10.1371/journal.pone.0069422 23922709 PMC3726668

[B33] Dacks A. M. Riffell J. A. Martin J. P. Gage S. L. Nighorn A. J. (2012a). Olfactory modulation by dopamine in the context of aversive learning. J. Neurophysiol. 108, 539–550. 10.1152/jn.00159.2012 22552185 PMC3404788

[B34] Dacks A. M. Siniscalchi M. J. Weiss K. R. (2012b). Removal of default state-associated inhibition during repetition priming improves response articulation. J. Neurosci. 32, 17740–17752. 10.1523/JNEUROSCI.4137-12.2012 23223294 PMC3529184

[B35] Dayan P. Huys Q. (2015). Serotonin’s many meanings elude simple theories. Elife 4:e07390. 10.7554/eLife.07390 25853523 PMC4389267

[B36] Ennis M. Zhou F. M. Ciombor K. J. Aroniadou-Anderjaska V. Hayar A. Borrelli E.. (2001). Dopamine D2 receptor-mediated presynaptic inhibition of olfactory nerve terminals. J. Neurophysiol. 86, 2986–2997. 10.1152/jn.2001.86.6.2986 11731555

[B37] Eyre M. D. Antal M. Nusser Z. (2008). Distinct deep short-axon cell subtypes of the main olfactory bulb provide novel intrabulbar and extrabulbar GABAergic connections. J. Neurosci. 28, 8217–8229. 10.1523/JNEUROSCI.2490-08.2008 18701684 PMC2630517

[B38] Fletcher M. L. Chen W. R. (2010). Neural correlates of olfactory learning: critical role of centrifugal neuromodulation. Learn. Mem. 17, 561–570. 10.1101/lm.941510 20980444 PMC2981412

[B39] Friedman A. K. Weiss K. R. (2010). Repetition priming of motoneuronal activity in a small motor network: intercellular and intracellular signaling. J. Neurosci. 30, 8906–8919. 10.1523/JNEUROSCI.1287-10.2010 20592213 PMC2914619

[B40] Fusca D. Schachtner J. Kloppenburg P. (2015). Colocalization of allatotropin and tachykinin-related peptides with classical transmitters in physiologically distinct subtypes of olfactory local interneurons in the cockroach (*Periplaneta americana*). J. Comp. Neurol. 523, 1569–1586. 10.1002/cne.23757 25678036

[B41] Gall C. Seroogy K. B. Brecha N. (1986). Distribution of VIP- and NPY-like immunoreactivities in rat main olfactory bulb. Brain Res. 374, 389–394. 10.1016/0006-8993(86)90436-1 2424562

[B42] Gatellier L. Nagao T. Kanzaki R. (2004). Serotonin modifies the sensitivity of the male silkmoth to pheromone. J. Exp. Biol. 207, 2487–2496. 10.1242/jeb.01035 15184520

[B43] Goldman A. L. Van der Goes van Naters W. Lessing D. Warr C. G. Carlson J. R. (2005). Coexpression of two functional odor receptors in one neuron. Neuron 45, 661–666. 10.1016/j.neuron.2005.01.025 15748842

[B44] Gómez C. Briñón J. G. Barbado M. V. Weruaga E. Valero J. Alonso J. R. (2005). Heterogeneous targeting of centrifugal inputs to the glomerular layer of the main olfactory bulb. J. Chem. Neuroanat. 29, 238–254. 10.1016/j.jchemneu.2005.01.005 15927786

[B45] Gupta N. Stopfer M. (2011). Insect olfactory coding and memory at multiple timescales. Curr. Opin. Neurobiol. 21, 768–773. 10.1016/j.conb.2011.05.005 21632235 PMC3182293

[B46] Hamanaka Y. Minoura R. Nishino H. Miura T. Mizunami M. (2016). Dopamine- and tyrosine hydroxylase-immunoreactive neurons in the brain of the american cockroach, *Periplaneta americana*. PLoS One 11:e0160531. 10.1371/journal.pone.0160531 27494326 PMC4975486

[B47] Hardy A. Palouzier-Paulignan B. Duchamp A. Royet J. P. Duchamp-Viret P. (2005). 5-Hydroxytryptamine action in the rat olfactory bulb: *in vitro* electrophysiological patch-clamp recordings of juxtaglomerular and mitral cells. Neuroscience 131, 717–731. 10.1016/j.neuroscience.2004.10.034 15730876

[B48] Harris-Warrick R. M. Marder E. (1991). Modulation of neural networks for behavior. Annu. Rev. Neurosci. 14, 39–57. 10.1146/annurev.neuro.14.1.39 2031576

[B49] Hayashi K. Nakao K. Nakamura K. (2015). Appetitive and aversive information coding in the primate dorsal raphé nucleus. J. Neurosci. 35, 6195–6208. 10.1523/JNEUROSCI.2860-14.2015 25878290 PMC6605165

[B50] Hellendall R. P. Schambra U. B. Liu J. P. Lauder J. M. (1993). Prenatal expression of 5-HT_1C_ and 5-HT_2_ receptors in the rat central nervous system. Exp. Neurol. 120, 186–201. 10.1006/exnr.1993.1054 8491279

[B51] Hildebrand J. G. Shepherd G. M. (1997). Mechanisms of olfactory discrimination: converging evidence for common principles across phyla. Annu. Rev. Neurosci. 20, 595–631. 10.1146/annurev.neuro.20.1.595 9056726

[B52] Homberg U. Kingan T. G. Hildebrand J. G. (1990). Distribution of FMRFamide-like immunoreactivity in the brain and suboesophageal ganglion of the sphinx moth Manduca sexta and colocalization with SCPB-, BPP-, and GABA-like immunoreactivity. Cell Tissue Res. 259, 401–419. 10.1007/bf01740767 2180574

[B53] Hsia A. Y. Vincent J. D. Lledo P. M. (1999). Dopamine depresses synaptic inputs into the olfactory bulb. J. Neurophysiol. 82, 1082–1085. 10444702 10.1152/jn.1999.82.2.1082

[B54] Huang Z. Thiebaud N. Fadool D. A. (2017). Differential serotonergic modulation across the main and accessory olfactory bulbs. J. Physiol. 595, 3515–3533. 10.1113/JP273945 28229459 PMC5451723

[B55] Ignell R. (2001). Monoamines and neuropeptides in antennal lobe interneurons of the desert locust, Schistocerca gregana: an immunocytochemical study. Cell Tissue Res. 306, 143–156. 10.1007/s004410100434 11683175

[B56] Ignell R. Root C. M. Birse R. T. Wang J. W. Nässel D. R. Winther A. M. (2009). Presynaptic peptidergic modulation of olfactory receptor neurons in *Drosophila*. Proc. Natl. Acad. Sci. U S A 106, 13070–13075. 10.1073/pnas.0813004106 19625621 PMC2722350

[B57] Jacobs B. L. Fornal C. A. (1991). Activity of brain serotonergic neurons in the behaving animal. Pharmacol. Rev. 43, 563–578. 1775508

[B58] Joseph R. M. Carlson J. R. (2015). *Drosophila* chemoreceptors: a molecular interface between the chemical world and the brain. Trends Genet. 31, 683–695. 10.1016/j.tig.2015.09.005 26477743 PMC4674303

[B59] Kaczmarek L. K. Levitan I. B. (1987). Neuromodulation: The Biochemical Control of Neuronal Excitability. New York, NY: Oxford University Press.

[B60] Kapoor V. Provost A. C. Agarwal P. Murthy V. N. (2016). Activation of raphe nuclei triggers rapid and distinct effects on parallel olfactory bulb output channels. Nat. Neurosci. 19, 271–282. 10.1038/nn.4219 26752161 PMC4948943

[B61] Katz P. S. (1995). Intrinsic and extrinsic neuromodulation of motor circuits. Curr. Opin. Neurobiol. 5, 799–808. 10.1016/0959-4388(95)80109-x 8805409

[B62] Katz P. S. (1999). Beyond Neurotransmission: Neuromodulation and its Importance for Information Processing. New York, NY: Oxford University Press.

[B63] Katz P. S. Frost W. N. (1995). Intrinsic neuromodulation in the Tritonia swim CPG: serotonin mediates both neuromodulation and neurotransmission by the dorsal swim interneurons. J. Neurophysiol. 74, 2281–2294. 8747191 10.1152/jn.1995.74.6.2281

[B64] Katz P. S. Frost W. N. (1996). Intrinsic neuromodulation: altering neuronal circuits from within. Trends Neurosci. 19, 54–61. 10.1016/0166-2236(97)90029-1 8820868

[B65] Kent K. S. Hoskins S. G. Hildebrand J. G. (1987). A novel serotonin-immunoreactive neuron in the antennal lobe of the sphinx moth Manduca sexta persists throughout postembryonic life. J. Neurobiol. 18, 451–465. 10.1002/neu.480180506 3309187

[B66] Kirchhof B. S. Homberg U. Mercer A. R. (1999). Development of dopamine-immunoreactive neurons associated with the antennal lobes of the honey bee, Apis mellifera. J. Comp. Neurol. 411, 643–653. 10.1002/(sici)1096-9861(19990906)411:4<643::aid-cne8>3.0.co;2-o 10421873

[B67] Kiselycznyk C. L. Zhang S. Linster C. (2006). Role of centrifugal projections to the olfactory bulb in olfactory processing. Learn. Mem. 13, 575–579. 10.1101/lm.285706 16980549

[B68] Kiyokage E. Pan Y. Z. Shao Z. Kobayashi K. Szabo G. Yanagawa Y.. (2010). Molecular identity of periglomerular and short axon cells. J. Neurosci. 30, 1185–1196. 10.1523/JNEUROSCI.3497-09.2010 20089927 PMC3718026

[B69] Kloppenburg P. Ferns D. Mercer A. R. (1999). Serotonin enhances central olfactory neuron responses to female sex pheromone in the male sphinx moth manduca sexta. J. Neurosci. 19, 8172–8181. 10493719 10.1523/JNEUROSCI.19-19-08172.1999 PMC6783045

[B70] Kloppenburg P. Mercer A. R. (2008). Serotonin modulation of moth central olfactory neurons. Annu. Rev. Entomol. 53, 179–190. 10.1146/annurev.ento.53.103106.093408 18067443

[B71] Ko K. I. Root C. M. Lindsay S. A. Zaninovich O. A. Shepherd A. K. Wasserman S. A.. (2015). Starvation promotes concerted modulation of appetitive olfactory behavior via parallel neuromodulatory circuits. Elife 4:e08298. 10.7554/eLife.08298 26208339 PMC4531282

[B72] Koh H. Y. Weiss K. R. (2005). Peptidergic contribution to posttetanic potentiation at a central synapse of aplysia. J. Neurophysiol. 94, 1281–1286. 10.1152/jn.00073.2005 15817651

[B73] Koh H. Y. Weiss K. R. (2007). Activity-dependent peptidergic modulation of the plateau-generating neuron B64 in the feeding network of Aplysia. J. Neurophysiol. 97, 1862–1867. 10.1152/jn.01230.2006 17202238

[B74] Kosaka T. Kosaka K. (2016). Neuronal organization of the main olfactory bulb revisited. Anat. Sci. Int. 91, 115–127. 10.1007/s12565-015-0309-7 26514846

[B75] Koster N. L. Norman A. B. Richtand N. M. Nickell W. T. Puche A. C. Pixley S. K.. (1999). Olfactory receptor neurons express D2 dopamine receptors. J. Comp. Neurol. 411, 666–673. 10.1002/(sici)1096-9861(19990906)411:4<666::aid-cne10>3.0.co;2-s 10421875

[B76] Kupfermann I. (1974). Feeding behavior in Aplysia: a simple system for the study of motivation. Behav. Biol. 10, 1–26. 10.1016/s0091-6773(74)91644-7 4815142

[B77] Kupfermann I. (1979). Modulatory actions of neurotransmitters. Annu. Rev. Neurosci. 2, 447–465. 10.1146/annurev.ne.02.030179.002311 44174

[B78] Kupfermann I. Weiss K. R. (1982). Activity of an identified serotonergic neuron in free moving Aplysia correlates with behavioral arousal. Brain Res. 241, 334–337. 10.1016/0006-8993(82)91072-1 7104716

[B79] Langdon P. E. Harley C. W. McLean J. H. (1997). Increased β adrenoceptor activation overcomes conditioned olfactory learning deficits induced by serotonin depletion. Dev. Brain Res. 102, 291–293. 10.1016/s0165-3806(97)00090-4 9352112

[B80] Lei H. Christensen T. A. Hildebrand J. G. (2002). Local inhibition modulates odor-evoked synchronization of glomerulus-specific output neurons. Nat. Neurosci. 5, 557–565. 10.1038/nn859 12006983

[B81] Li Y. Zhong W. Wang D. Feng Q. Liu Z. Zhou J.. (2016). Serotonin neurons in the dorsal raphe nucleus encode reward signals. Nat. Commun. 7:10503. 10.1038/ncomms10503 26818705 PMC4738365

[B82] Linn C. E. Roelofs W. L. (1986). Modulatory effects of octopamine and serotonin on male sensitivity and periodicity of response to sex-pheromone in the cabbage-looper moth, trichoplusia-ni. Arch. Insect Biochem. Physiol. 3, 161–171. 10.1002/arch.940030206

[B83] Linster C. Cleland T. A. (2002). Cholinergic modulation of sensory representations in the olfactory bulb. Neural Netw. 15, 709–717. 10.1016/s0893-6080(02)00061-8 12371521

[B84] Linster C. Cleland T. A. (2016). Neuromodulation of olfactory transformations. Curr. Opin. Neurobiol. 40, 170–177. 10.1016/j.conb.2016.07.006 27564660

[B85] Liu S. Aungst J. L. Puche A. C. Shipley M. T. (2012). Serotonin modulates the population activity profile of olfactory bulb external tufted cells. J. Neurophysiol. 107, 473–483. 10.1152/jn.00741.2011 22013233 PMC3349690

[B86] Liu S. Plachez C. Shao Z. Puche A. Shipley M. T. (2013). Olfactory bulb short axon cell release of GABA and dopamine produces a temporally biphasic inhibition-excitation response in external tufted cells. J. Neurosci. 33, 2916–2926. 10.1523/JNEUROSCI.3607-12.2013 23407950 PMC3727441

[B87] Liu Z. Zhou J. Li Y. Hu F. Lu Y. Ma M.. (2014). Dorsal raphe neurons signal reward through 5-HT and glutamate. Neuron 81, 1360–1374. 10.1016/j.neuron.2014.02.010 24656254 PMC4411946

[B88] Lizbinski K. M. Marsat G. Dacks A. M. (2017). Transmitter co-expression reveals key organizational principles of local interneuron heterogeneity in the olfactory system. BioRxiv

[B89] Lizbinski K. M. Metheny J. D. Bradley S. P. Kesari A. Dacks A. M. (2016). The anatomical basis for modulatory convergence in the antennal lobe of Manduca sexta. J. Comp. Neurol. 524, 1859–1875. 10.1002/cne.23926 26560074 PMC4833642

[B90] Lucaites V. L. Krushinski J. H. Schaus J. M. Audia J. E. Nelson D. L. (2005). [^3H^]LY334370, a novel radioligand for the 5-HT_1F_ receptor. II. Autoradiographic localization in rat, guinea pig, monkey and human brain. Naunyn Schmiedebergs. Arch. Pharmacol. 371, 178–184. 10.1007/s00210-005-1036-8 15900511

[B91] Luo M. Li Y. Zhong W. (2016). Do dorsal raphe 5-HT neurons encode “beneficialness”? Neurobiol. Learn. Mem. 135, 40–49. 10.1016/j.nlm.2016.08.008 27544850

[B92] MacLeod K. Laurent G. (1996). Distinct mechanisms for synchronization and temporal patterning of odor-encoding neural assemblies. Science 274, 976–979. 10.1126/science.274.5289.976 8875938

[B93] Mandairon N. Ferretti C. J. Stack C. M. Rubin D. B. Cleland T. A. Linster C. (2006). Cholinergic modulation in the olfactory bulb influences spontaneous olfactory discrimination in adult rats. Eur. J. Neurosci. 24, 3234–3244. 10.1111/j.1460-9568.2006.05212.x 17156384

[B94] Martin J. P. Beyerlein A. Dacks A. M. Reisenman C. E. Riffell J. A. Lei H.. (2011). The neurobiology of insect olfaction: sensory processing in a comparative context. Prog. Neurobiol. 95, 427–447. 10.1016/j.pneurobio.2011.09.007 21963552

[B95] Matsutani S. Yamamoto N. (2008). Centrifugal innervation of the mammalian olfactory bulb. Anat. Sci. Int. 83, 218–227. 10.1111/j.1447-073X.2007.00223.x 19159349

[B96] McGann J. P. (2013). Presynaptic inhibition of olfactory sensory neurons: new mechanisms and potential functions. Chem. Senses 38, 459–474. 10.1093/chemse/bjt018 23761680 PMC3685425

[B97] McGann J. P. Pirez N. Gainey M. A. Muratore C. Elias A. S. Wachowiak M. (2005). Odorant representations are modulated by intra- but not interglomerular presynaptic inhibition of olfactory sensory neurons. Neuron 48, 1039–1053. 10.1016/j.neuron.2005.10.031 16364906

[B98] McLean J. H. Darby-King A. Hodge E. (1996). 5-HT_2_ receptor involvement in conditioned olfactory learning in the neonate rat pup. Behav. Neurosci. 110, 1426–1434. 10.1037/0735-7044.110.6.1426 8986343

[B99] McLean J. H. Darby-King A. Paterno G. D. (1995). Localization of 5-HT_2A_ receptor mRNA by *in situ* hybridization in the olfactory bulb of the postnatal rat. J. Comp. Neurol. 353, 371–378. 10.1002/cne.903530305 7751437

[B100] McLean J. H. Darby-King A. Sullivan R. M. King S. R. (1993). Serotonergic influence on olfactory learning in the neonate rat. Behav. Neural Biol. 60, 152–162. 10.1016/0163-1047(93)90257-i 7906939

[B101] McLean J. H. Shipley M. T. (1987). Serotonergic afferents to the rat olfactory bulb: I. Origins and laminar specificity of serotonergic inputs in the adult rat. J. Neurosci. 7, 3016–3028. 2822862 10.1523/JNEUROSCI.07-10-03016.1987 PMC6569188

[B102] Mercer A. R. Hayashi J. H. Hildebrand J. G. (1995). Modulatory effects of 5-hydroxytryptamine on voltage-activated currents in cultured antennal lobe neurones of the sphinx moth Manduca sexta. J. Exp. Biol. 198, 613–627. 7714451 10.1242/jeb.198.3.613

[B103] Mercer A. R. Kloppenburg P. Hildebrand J. G. (1996). Serotonin-induced changes in the excitability of cultured antennal-lobe neurons of the sphinx moth Manduca sexta. J. Comp. Physiol. A 178, 21–31. 10.1007/bf00189587 8568722

[B104] Miyazaki K. Miyazaki K. W. Doya K. (2011a). Activation of dorsal raphe serotonin neurons underlies waiting for delayed rewards. J. Neurosci. 31, 469–479. 10.1523/JNEUROSCI.3714-10.2011 21228157 PMC6623450

[B105] Miyazaki K. W. Miyazaki K. Doya K. (2011b). Activation of the central serotonergic system in response to delayed but not omitted rewards. Eur. J. Neurosci. 33, 153–160. 10.1111/j.1460-9568.2010.07480.x 21070390 PMC3040841

[B106] Morgan P. T. Perrins R. Lloyd P. E. Weiss K. R. (2000). Intrinsic and extrinsic modulation of a single central pattern generating circuit. J. Neurophysiol. 84, 1186–1193. 10.1152/jn.2000.84.3.1186 10979994

[B107] Mouret A. Murray K. Lledo P. M. (2009). Centrifugal drive onto local inhibitory interneurons of the olfactory bulb. Ann. N Y Acad. Sci. 1170, 239–254. 10.1111/j.1749-6632.2009.03913.x 19686142

[B108] Murphy G. J. Glickfeld L. L. Balsen Z. Isaacson J. S. (2004). Sensory neuron signaling to the brain: properties of transmitter release from olfactory nerve terminals. J. Neurosci. 24, 3023–3030. 10.1523/JNEUROSCI.5745-03.2004 15044541 PMC6729835

[B109] Muzerelle A. Scotto-Lomassese S. Bernard J. F. Soiza-Reilly M. Gaspar P. (2016). Conditional anterograde tracing reveals distinct targeting of individual serotonin cell groups (B5–B9) to the forebrain and brainstem. Brain Struct. Funct. 221, 535–561. 10.1007/s00429-014-0924-4 25403254 PMC4750555

[B110] Nagayama S. Homma R. Imamura F. (2014). Neuronal organization of olfactory bulb circuits. Front. Neural Circuits 8:98. 10.3389/fncir.2014.00098 25232305 PMC4153298

[B111] Nakamura K. (2013). The role of the dorsal raphe nucleus in reward-seeking behavior. Front. Integr. Neurosci. 7:60. 10.3389/fnint.2013.00060 23986662 PMC3753458

[B112] Nakamura K. Matsumoto M. Hikosaka O. (2008). Reward-dependent modulation of neuronal activity in the primate dorsal raphe nucleus. J. Neurosci. 28, 5331–5343. 10.1523/JNEUROSCI.0021-08.2008 18480289 PMC3329731

[B113] Nickell W. T. Behbehani M. M. Shipley M. T. (1994). Evidence for GABA_B_-mediated inhibition of transmission from the olfactory nerve to mitral cells in the rat olfactory bulb. Brain Res. Bull. 35, 119–123. 10.1016/0361-9230(94)90091-4 7953767

[B114] Okaty B. W. Freret M. E. Rood B. D. Brust R. D. Hennessy M. L. deBairos D.. (2015). Multi-scale molecular deconstruction of the serotonin neuron system. Neuron 88, 774–791. 10.1016/j.neuron.2015.10.007 26549332 PMC4809055

[B115] Olsen S. R. Bhandawat V. Wilson R. I. (2010). Divisive normalization in olfactory population codes. Neuron 66, 287–299. 10.1016/j.neuron.2010.04.009 20435004 PMC2866644

[B116] Olsen S. R. Wilson R. I. (2008). Lateral presynaptic inhibition mediates gain control in an olfactory circuit. Nature 452, 956–960. 10.1038/nature06864 18344978 PMC2824883

[B117] Owald D. Waddell S. (2015). Olfactory learning skews mushroom body output pathways to steer behavioral choice in *Drosophila*. Curr. Opin. Neurobiol. 35, 178–184. 10.1016/j.conb.2015.10.002 26496148 PMC4835525

[B118] Park S. P. Lopez-Rodriguez F. Wilson C. L. Maidment N. Matsumoto Y. Engel J. Jr. (1999). *In vivo* microdialysis measures of extracellular serotonin in the rat hippocampus during sleep-wakefulness. Brain Res. 833, 291–296. 10.1016/s0006-8993(99)01511-5 10375707

[B119] Petzold G. C. Hagiwara A. Murthy V. N. (2009). Serotonergic modulation of odor input to the mammalian olfactory bulb. Nat. Neurosci. 12, 784–791. 10.1038/nn.2335 19430472

[B120] Pinching A. J. Powell T. P. (1971). The neuropil of the periglomerular region of the olfactory bulb. J. Cell Sci. 9, 379–409. 5124504 10.1242/jcs.9.2.379

[B121] Pirez N. Wachowiak M. (2008). *In vivo* modulation of sensory input to the olfactory bulb by tonic and activity-dependent presynaptic inhibition of receptor neurons. J. Neurosci. 28, 6360–6371. 10.1523/JNEUROSCI.0793-08.2008 18562606 PMC2566846

[B122] Pollak Dorocic I. Fürth D. Xuan Y. Johansson Y. Pozzi L. Silberberg G.. (2014). A whole-brain atlas of inputs to serotonergic neurons of the dorsal and median raphe nuclei. Neuron 83, 663–678. 10.1016/j.neuron.2014.07.002 25102561

[B123] Price T. L. Darby-King A. Harley C. W. McLean J. H. (1998). Serotonin plays a permissive role in conditioned olfactory learning induced by norepinephrine in the neonate rat. Behav. Neurosci. 112, 1430–1437. 10.1037/0735-7044.112.6.1430 9926825

[B124] Proekt A. Weiss K. R. (2003). Convergent mechanisms mediate preparatory states and repetition priming in the feeding network of Aplysia. J. Neurosci. 23, 4029–4033. 12764089 10.1523/JNEUROSCI.23-10-04029.2003 PMC6741109

[B125] Ranade S. P. Mainen Z. F. (2009). Transient firing of dorsal raphe neurons encodes diverse and specific sensory, motor, and reward events. J. Neurophysiol. 102, 3026–3037. 10.1152/jn.00507.2009 19710375

[B126] Rehder V. Bicker G. Hammer M. (1987). Serotonin-immunoreactive neurons in the antennal lobes and suboesophageal ganglion of the honeybee. Cell Tissue Res. 247, 59–66. 10.1007/bf00216547

[B127] Reisenman C. E. Dacks A. M. Hildebrand J. G. (2011). Local interneuron diversity in the primary olfactory center of the moth Manduca sexta. J. Comp. Physiol. A Neuroethol. Sens. Neural Behav. Physiol. 197, 653–665. 10.1007/s00359-011-0625-x 21286727

[B128] Robinson B. L. McAlpine D. (2009). Gain control mechanisms in the auditory pathway. Curr. Opin. Neurobiol. 19, 402–407. 10.1016/j.conb.2009.07.006 19665367

[B129] Root C. M. Ko K. I. Jafari A. Wang J. W. (2011). Presynaptic facilitation by neuropeptide signaling mediates odor-driven food search. Cell 145, 133–144. 10.1016/j.cell.2011.02.008 21458672 PMC3073827

[B130] Root C. M. Masuyama K. Green D. S. Enell L. E. Nässel D. R. Lee C. H.. (2008). A presynaptic gain control mechanism fine-tunes olfactory behavior. Neuron 59, 311–321. 10.1016/j.neuron.2008.07.003 18667158 PMC2539065

[B131] Rosen S. C. Weiss K. R. Goldstein R. S. Kupfermann I. (1989). The role of a modulatory neuron in feeding and satiation in Aplysia: effects of lesioning of the serotonergic metacerebral cells. J. Neurosci. 9, 1562–1578. 2723741 10.1523/JNEUROSCI.09-05-01562.1989 PMC6569832

[B132] Roy B. Singh A. P. Shetty C. Chaudhary V. North A. Landgraf M.. (2007). Metamorphosis of an identified serotonergic neuron in the *Drosophila* olfactory system. Neural Dev. 2:20. 10.1186/1749-8104-2-20 17958902 PMC2129096

[B133] Saalmann Y. B. Kastner S. (2009). Gain control in the visual thalamus during perception and cognition. Curr. Opin. Neurobiol. 19, 408–414. 10.1016/j.conb.2009.05.007 19556121 PMC3140205

[B134] Sachse S. Beshel J. (2016). The good, the bad, and the hungry: how the central brain codes odor valence to facilitate food approach in *Drosophila*. Curr. Opin. Neurobiol. 40, 53–58. 10.1016/j.conb.2016.06.012 27393869 PMC5056820

[B135] Salecker I. Distler P. (1990). Serotonin-immunoreactive neurons in the antennal lobes of the American cockroach *Periplaneta americana*: light- and electron-microscopic observations. Histochemistry 94, 463–473. 10.1007/bf00272608 2283309

[B136] Schmidt L. J. Strowbridge B. W. (2014). Modulation of olfactory bulb network activity by serotonin: synchronous inhibition of mitral cells mediated by spatially localized GABAergic microcircuits. Learn. Mem. 21, 406–416. 10.1101/lm.035659.114 25031366 PMC4105717

[B137] Schultzhaus J. N. Saleem S. Iftikhar H. Carney G. E. (2017). The role of the *Drosophila* lateral horn in olfactory information processing and behavioral response. J. Insect Physiol. 98, 29–37. 10.1016/j.jinsphys.2016.11.007 27871975

[B138] Seki Y. Kanzaki R. (2008). Comprehensive morphological identification and GABA immunocytochemistry of antennal lobe local interneurons in Bombyx mori. J. Comp. Neurol. 506, 93–107. 10.1002/cne.21528 17990273

[B139] Seki Y. Rybak J. Wicher D. Sachse S. Hansson B. S. (2010). Physiological and morphological characterization of local interneurons in the *Drosophila* antennal lobe. J. Neurophysiol. 104, 1007–1019. 10.1152/jn.00249.2010 20505124

[B140] Seroogy K. B. Brecha N. Gall C. (1985). Distribution of cholecystokinin-like immunoreactivity in the rat main olfactory bulb. J. Comp. Neurol. 239, 373–383. 10.1002/cne.902390403 2864364

[B141] Shen Y. Monsma F. J. Jr. Metcalf M. A. Jose P. A. Hamblin M. W. Sibley D. R. (1993). Molecular cloning and expression of a 5-hydroxytryptamine7 serotonin receptor subtype. J. Biol. Chem. 268, 18200–18204. 8394362

[B142] Shepherd G. M. Chen W. R. Willhite D. Migliore M. Greer C. A. (2007). The olfactory granule cell: from classical enigma to central role in olfactory processing. Brain Res. Rev. 55, 373–382. 10.1016/j.brainresrev.2007.03.005 17434592

[B143] Shipley M. T. Ennis M. (1996). Functional organization of olfactory system. J. Neurobiol. 30, 123–176. 10.1002/(sici)1097-4695(199605)30:1<123::aid-neu11>3.3.co;2-s 8727988

[B144] Siju K. P. Reifenrath A. Scheiblich H. Neupert S. Predel R. Hansson B. S.. (2014). Neuropeptides in the antennal lobe of the yellow fever mosquito, Aedes aegypti. J. Comp. Neurol. 522, 592–608. 10.1002/cne.23434 23897410 PMC4265797

[B145] Sinakevitch I. Niwa M. Strausfeld N. J. (2005). Octopamine-like immunoreactivity in the honey bee and cockroach: comparable organization in the brain and subesophageal ganglion. J. Comp. Neurol. 488, 233–254. 10.1002/cne.20572 15952163

[B146] Sinakevitch I. Strausfeld N. J. (2006). Comparison of octopamine-like immunoreactivity in the brains of the fruit fly and blow fly. J. Comp. Neurol. 494, 460–475. 10.1002/cne.20799 16320256

[B147] Singh A. P. Das R. N. Rao G. Aggarwal A. Diegelmann S. Evers J. F.. (2013). Sensory neuron-derived eph regulates glomerular arbors and modulatory function of a central serotonergic neuron. PLoS Genet. 9:e1003452. 10.1371/journal.pgen.1003452 23637622 PMC3630106

[B148] Sizemore T. R. Dacks A. M. (2016). Serotonergic modulation differentially targets distinct network elements within the antennal lobe of *Drosophila melanogaster*. Sci. Rep. 6:37119. 10.1038/srep37119 27845422 PMC5109230

[B149] Steinfeld R. Herb J. T. Sprengel R. Schaefer A. T. Fukunaga I. (2015). Divergent innervation of the olfactory bulb by distinct raphe nuclei. J. Comp. Neurol. 523, 805–813. 10.1002/cne.23713 25420775 PMC4328392

[B150] Sun X. J. Tolbert L. P. Hildebrand J. G. (1993). Ramification pattern and ultrastructural characteristics of the serotonin-immunoreactive neuron in the antennal lobe of the moth Manduca sexta: a laser scanning confocal and electron microscopic study. J. Comp. Neurol. 338, 5–16. 10.1002/cne.903380103 8300899

[B151] Suzuki Y. Kiyokage E. Sohn J. Hioki H. Toida K. (2015). Structural basis for serotonergic regulation of neural circuits in the mouse olfactory bulb. J. Comp. Neurol. 523, 262–280. 10.1002/cne.23680 25234191

[B152] Tecott L. H. Maricq A. V. Julius D. (1993). Nervous system distribution of the serotonin 5-HT_3_ receptor mRNA. Proc. Natl. Acad. Sci. U S A 90, 1430–1434. 10.1073/pnas.90.4.1430 8434003 PMC45887

[B153] Tedjakumala S. R. Rouquette J. Boizeau M. L. Mesce K. A. Hotier L. Massou I.. (2017). A tyrosine-hydroxylase characterization of dopaminergic neurons in the honey bee brain. Front. Syst. Neurosci. 11:47. 10.3389/fnsys.2017.00047 28740466 PMC5502285

[B154] Trulson M. E. Jacobs B. L. (1979). Raphe unit activity in freely moving cats: correlation with level of behavioral arousal. Brain Res. 163, 135–150. 10.1016/0006-8993(79)90157-4 218676

[B155] Utz S. Huetteroth W. Vömel M. Schachtner J. (2008). Mas-allatotropin in the developing antennal lobe of the sphinx moth Manduca sexta: distribution, time course, developmental regulation, and colocalization with other neuropeptides. Dev. Neurobiol. 68, 123–142. 10.1002/dneu.20579 17948246

[B156] Vaaga C. E. Yorgason J. T. Williams J. T. Westbrook G. L. (2017). Presynaptic gain control by endogenous cotransmission of dopamine and GABA in the olfactory bulb. J. Neurophysiol. 117, 1163–1170. 10.1152/jn.00694.2016 28031402 PMC5340883

[B157] Vosshall L. B. (2000). Olfaction in *Drosophila*. Curr. Opin. Neurobiol. 10, 498–503. 10.1016/S0959-4388(00)00111-2 10981620

[B158] Vosshall L. B. Amrein H. Morozov P. S. Rzhetsky A. Axel R. (1999). A spatial map of olfactory receptor expression in the *Drosophila* antenna. Cell 96, 725–736. 10.1016/s0092-8674(00)80582-6 10089887

[B159] Vučinić D. Cohen L. B. Kosmidis E. K. (2006). Interglomerular center-surround inhibition shapes odorant-evoked input to the mouse olfactory bulb *in vivo*. J. Neurophysiol. 95, 1881–1887. 10.1152/jn.00918.2005 16319205

[B160] Wachowiak M. Cohen L. B. (1998). Presynaptic afferent inhibition of lobster olfactory receptor cells: reduced action-potential propagation into axon terminals. J. Neurophysiol. 80, 1011–1015. 9705490 10.1152/jn.1998.80.2.1011

[B161] Wachowiak M. Cohen L. B. (1999). Presynaptic inhibition of primary olfactory afferents mediated by different mechanisms in lobster and turtle. J. Neurosci. 19, 8808–8817. 10516300 10.1523/JNEUROSCI.19-20-08808.1999 PMC6782745

[B162] Wachowiak M. McGann J. P. Heyward P. M. Shao Z. Puche A. C. Shipley M. T. (2005). Inhibition [corrected] of olfactory receptor neuron input to olfactory bulb glomeruli mediated by suppression of presynaptic calcium influx. J. Neurophysiol. 94, 2700–2712. 10.1152/jn.00286.2005 15917320 PMC1282456

[B163] Wachowiak M. Shipley M. T. (2006). Coding and synaptic processing of sensory information in the glomerular layer of the olfactory bulb. Semin. Cell Dev. Biol. 17, 411–423. 10.1016/j.semcdb.2006.04.007 16765614

[B164] Waeber C. Grailhe R. Yu X. J. Hen R. Moskowitz M. A. (1998). Putative 5-ht_5_ receptors: localization in the mouse CNS and lack of effect in the inhibition of dural protein extravasation. Ann. N Y Acad. Sci. 861, 85–90. 10.1111/j.1749-6632.1998.tb10177.x 9928243

[B165] Waldrop B. Christensen T. A. Hildebrand J. G. (1987). GABA-mediated synaptic inhibition of projection neurons in the antennal lobes of the sphinx moth, Manduca sexta. J. Comp. Physiol. A 161, 23–32. 10.1007/bf00609452 3039128

[B166] Wang J. W. (2012). Presynaptic modulation of early olfactory processing in *Drosophila*. Dev. Neurobiol. 72, 87–99. 10.1002/dneu.20936 21688402 PMC3246013

[B167] Wasserman S. Salomon A. Frye M. A. (2013). *Drosophila* tracks carbon dioxide in flight. Curr. Biol. 23, 301–306. 10.1016/j.cub.2012.12.03 23352695 PMC3810385

[B168] Watts S. W. Gackenheimer S. L. Gehlert D. R. Cohen M. L. (1994). Autoradiographic comparison of [^125^I]LSD-labeled 5-HT_2A_ receptor distribution in rat and guinea pig brain. Neurochem. Int. 24, 565–574. 10.1016/0197-0186(94)90009-4 7981639

[B169] Wegerhoff R. (1999). GABA and serotonin immunoreactivity during postembryonic brain development in the beetle Tenebrio molitor. Microsc. Res. Tech. 45, 154–164. 10.1002/(sici)1097-0029(19990501)45:3<154::aid-jemt3>3.0.co;2-5 10344767

[B170] Weissbourd B. Ren J. DeLoach K. E. Guenthner C. J. Miyamichi K. Luo L. (2014). Presynaptic partners of dorsal raphe serotonergic and GABAergic neurons. Neuron 83, 645–662. 10.1016/j.neuron.2014.06.024 25102560 PMC4779447

[B172] Wilson R. I. (2013). Early olfactory processing in *Drosophila*: mechanisms and principles. Annu. Rev. Neurosci. 36, 217–241. 10.1146/annurev-neuro-062111-150533 23841839 PMC3933953

[B173] Wilson R. I. Laurent G. (2005). Role of GABAergic inhibition in shaping odor-evoked spatiotemporal patterns in the *Drosophila* antennal lobe. J. Neurosci. 25, 9069–9079. 10.1523/jneurosci.2070-05.2005 16207866 PMC6725763

[B174] Wilson R. I. Turner G. C. Laurent G. (2004). Transformation of olfactory representations in the *Drosophila* antennal lobe. Science 303, 366–370. 10.1126/science.1090782 14684826

[B175] Won M. H. Ohno T. Suh J. G. Lee J. C. Jo S. M. Oh Y. S.. (1998). Serotonergic neurons are present and innervate blood vessels in the olfactory bulb of the laboratory shrew, *Suncus murinus*. Neurosci. Lett. 243, 53–56. 10.1016/s0304-3940(98)00084-6 9535111

[B176] Wright D. E. Seroogy K. B. Lundgren K. H. Davis B. M. Jennes L. (1995). Comparative localization of serotonin_1A, 1C_, and _2_ receptor subtype mRNAs in rat brain. J. Comp. Neurol. 351, 357–373. 10.1002/cne.903510304 7706547

[B177] Xu L. He J. Kaiser A. Gräber N. Schläger L. Ritze Y.. (2016). A single pair of serotonergic neurons counteracts serotonergic inhibition of ethanol attraction in *Drosophila*. PLoS One 11:e0167518. 10.1371/journal.pone.0167518 27936023 PMC5147910

[B178] Yuan Q. Harley C. W. McLean J. H. (2003). Mitral cell β1 and 5-HT_2A_ receptor colocalization and cAMP coregulation: a new model of norepinephrine-induced learning in the olfactory bulb. Learn. Mem. 10, 5–15. 10.1101/lm.54803 12551959 PMC196649

[B179] Zars T. (2000). Behavioral functions of the insect mushroom bodies. Curr. Opin. Neurobiol. 10, 790–795. 10.1016/s0959-4388(00)00147-1 11240291

[B180] Zhang X. Gaudry Q. (2016). Functional integration of a serotonergic neuron in the *Drosophila* antennal lobe. Elife 5:e16836. 10.7554/eLife.16836 27572257 PMC5030083

[B171] Zhong W. Li Y. Feng Q. Luo M. (2017). Learning and stress shape the reward response patterns of serotonin neurons. J. Neurosci. 37, 8863–8875. 10.1523/JNEUROSCI.1181-17.2017 28821671 PMC6596795

